# Current status of intestinal parasitosis among patients attending teaching hospitals in Zagazig district, Northeastern Egypt

**DOI:** 10.1007/s00436-022-07500-z

**Published:** 2022-04-01

**Authors:** Marwa Omar, Heba O. Abdelal

**Affiliations:** 1 Department of Medical Parasitology, Faculty of Medicine, Zagazig University, Sharkia Governorate, Gameyet Almohafza St. 1, Menya Al Kamh, City of Zagazig, 44511 Egypt; 2 LIS: Cross-National Data Center, Maison des Sciences Humaines - 5e étage, 11- porte des Sciences, L-4366 Esch-Belval, Luxembourg

**Keywords:** Parasites, Egypt, Mini-FLOTAC, Prevalence, Mini-Parasep®

## Abstract

Almost 80% of health problems in the developing world are due to malnutrition and infectious diseases, which are mainly parasitic. Updated records on the prevalence of parasitic infections and the potential risk factors are essential to enhancing control strategies. Therefore, this study was conducted to evaluate the current situation of intestinal parasitism among patients attending teaching hospitals in Zagazig district, Northeastern Egypt. The study involved five hundred cases. They were all subjected to faecal examination using direct smear measure and two commercial faecal concentrators: Mini-Parasep® solvent-free and Mini-FLOTAC procedures. Mini-FLOTAC was performed with two solutions (FS2: saturated sodium chloride and FS7: zinc sulphate). The overall prevalence of intestinal parasitic infections was 56%. Different species were identified, like *Giardia lamblia* (12.6%), *Entamoeba histolytica/dispar* (10%), *Ascaris lumbricoides* (8.8%) and *Hymenolepis nana* (8.6%). Data analyses revealed a significant association of intestinal parasitism with different socio-demographic features of the participants. Our results showed a better diagnostic performance of Mini-Parasep® in the overall recovery of intestinal parasites. It was more accurate than Mini-FLOTAC in diagnosing both helminths and protozoan infections. Mini-FLOTAC (FS2) exhibited a higher sensitivity than FS7 for helminth recovery (74.6% vs 53.4%), while FS7 was more sensitive for protozoan infections (50.6% vs 43.8%). Intestinal parasitosis remains a challenging health problem in Zagazig city, wherever reliable diagnostic approaches are limited. Thus, our study has proposed the value of the commercial concentrators (Mini-Parasep® and Mini-FLOTAC) as alternative techniques for diagnosing a large variety of parasite species in resource-constrained settings.

## Introduction

Intestinal parasitic infections (IPIs) are recognized as one of the neglected tropical diseases which constitute a global health concern, especially in developing countries (Taghipour et al. 2021). Over 3 billion people worldwide are infected with different intestinal parasites, causing morbidity in 450 million individuals (Hailegebriel 2017). The prevalence of these infections is governed by geographical, behavioural, biological and socioeconomic factors. They are closely associated with the humid tropical climate, limited access to clean water, poor environmental sanitation, overcrowding and low family income. All these conditions favour and facilitate the growth, transmission and exposure to intestinal parasites (Amer et al. 2018).

Ingestion of contaminated food and water is the commonest route for transmitting different intestinal parasites. Infection can also be acquired after active skin penetration by infective larval stages in polluted soil (Moses et al. 2013). Intestinal parasites are key aetiological agents of different gastrointestinal troubles such as dysentery, diarrhoea, abdominal distension, vomiting and lack of appetite. They can also cause iron deficiency anaemia, growth retardation and mentally related disorders. Such clinical complications commonly affect high-risk populations like pregnant females, immunocompromised individuals and children (Mahmud et al. 2020). Furthermore, intestinal parasitic diseases account for nearly 39 million disability-adjusted life years (DALYs) globally (Stephenson et al. 2000).

Despite the notable improvement in sanitation infrastructure and hygienic status, intestinal parasitism remains a significant health challenge in Egypt (Monib et al. 2016). Owing to the broad diversity in cultural, educational, geographic and socioeconomic status, reports on prevalence rates of intestinal parasites vary in different localities in Egypt. In the Greater Cairo region, about 51% of the patients complaining of gastrointestinal symptoms were positive for different intestinal parasitic infections (Hussein et al. 2017). In Sohag Governorate, Southern Egypt, El-Nadi et al. (2017) reported a higher infection rate (63.5%) among elementary school children. Thus, studies on the prevalence of IPIs and associated risk factors are essential to identify high-risk communities and improve different control measures (Abera et al. 2013).

In the field studies, the search for an optimal applicable diagnostic tool is a bit challenging. The choice of a specific technique is determined by its sensitivity, simplicity, affordability and cost (Garcia 2001). Thus, different commercial faecal parasite concentrators have been developed recently to replace the traditional concentration methods, particularly in low transmission settings (Saez et al. 2011). Mini-Parasep® solvent-free (SF) concentrator is a closed single-use device that removes the debris and fat from faecal samples through a two-stage filtration matrix without using chemical agents. Besides, it is a cost-effective tool that could be used in large-scale epidemiological surveys (Mohram et al. 2020). The Mini-FLOTAC method has also been recently introduced to provide laboratories in resource-limited facilities with a reliable measure for both diagnostic and epidemiological purposes. The main advantage of this technique is that it does not require any centrifugation steps, and so, it is practical in processing large numbers of samples that require a rapid diagnosis (Barda et al. 2013).

Several studies were conducted to evaluate the situation of intestinal parasitism in the city of Zagazig, Northeastern Egypt. Yet, most of these studies have focused on the distribution of specific parasites among specific populations, particularly children. For example, Farghly et al. (2016) denoted a prevalence rate of 21.07% for soil-transmitted helminths among school children. Also, Mohammad et al. (2021) reported a molecular prevalence of 25.5% for *Cryptosporidium* species among children suffering from malignancy. Unfortunately, there is limited data about the extent of different human intestinal parasites among different population segments in Zagazig city. Besides, the numerous risk factors which might aggravate the transmission of intestinal parasites are still overlooked. In line with this, our study aims to provide an updated report on the current profile of intestinal parasites and the potential risk factors among patients attending two principal teaching hospitals (Zagazig University Hospital and Al-Ahrar Teaching Hospital) in Zagazig district. Moreover, to the best of our knowledge, this is the first study that combined two commercial faecal concentrators (Mini-Parasep® SF and Mini-FLOTAC) as innovative diagnostic measures to assess the current situation of human intestinal parasitism in the city of Zagazig.

## Subjects and methods

### Study type and area

This cross-sectional prevalence study was carried out in the city of Zagazig, Sharqia Province, Northeastern Egypt, during the period from November 2019 to early April 2021. Zagazig is the capital of Sharqia Governorate which is situated about 80 km from Cairo to the Northeast. It is located at latitude 30.57° N and longitude 31.50° E. The total area of the urban boundary of Zagazig is 4275 acres, with a population of 481,500 in the year 2027 (El-Barmelgy and El-Khateb 2020). Participants in this study were selected from two main teaching hospitals in Zagazig district: Zagazig University Hospital, which is located 3.6 km from the centre of the city, and Al-Ahrar Teaching Hospital (2.0 km from the city centre).

### Study population and design

The participants in the present study aged between 11 and 60 years old. They were recruited from Pediatrics and Internal Medicine in- and outpatient Clinics of both Zagazig University and Al-Ahrar Teaching Hospitals. A simple random sampling method was employed to select the participants in the study. The cases consisted of 290 males (58%) and 210 females (42%), with symptoms suggestive of intestinal parasitic infections. Patients on anti-parasitic agents were excluded from the study. A semi-structured questionnaire was established to gather information on age, sex, hospitalized or non-hospitalized status, socioeconomic profile (family monthly income), type of residence, number of house rooms and number of individuals in the household of all studied cases. The questionnaires were accurately checked for completeness. In the current study, socioeconomic status (SES) was graded as low, medium and high according to Fahmy et al. (2015) grading scale. The household crowding index (HCI) was described as the total number of co-residents per household divided by the total number of rooms. It was grouped into three distinct categories as follows: the first category < 1; the second category, 1–2; and the third category, > 2 residents per room (Melki et al. 2004).

### Sample collection and examination

Faecal samples were collected in sterile, dry and labelled plastic containers. About 2–5 g of solid or 10 ml of liquid stool specimens was obtained from each patient. All parasitological procedures were conducted at the Medical Parasitology Department (Post-Graduate Research Laboratory), Faculty of Medicine, Zagazig University. Stool samples were examined promptly after collection. Each sample was processed as follows.

### Macroscopic examination

The stool specimens were examined macroscopically for consistency, colour and the presence of blood, mucous or adult intestinal worms.

### Microscopic examination

Each stool sample was divided into three equal parts and examined in parallel by three different procedures: direct smear measure, Mini-Parasep® solvent-free (SF) and Mini-FLOTAC commercial faecal concentrators. Formed and semi-formed specimens were measured by the sensitive gram scale, while liquid diarrheic samples were measured in millilitres using calibrated cylinders.

1. Direct smear examination was conducted using normal saline (0.9%), Lugol’s iodine and eosin methods**.** Several slides were prepared from each single sample. For each slide, about 2 mg (the size of a match-stick head) of stool was obtained from both the surface and the inside of the specimen to increase the rate of detection of different parasites (Cheesbrough 1991). Wet mount preparations were used for detecting helminth ova and protozoan cysts. Permanent stained smears were performed using the modified Ziehl–Neelsen (MZN) technique for detecting intestinal coccidian parasites (Utzinger et al. 2010).
2. The Mini-Parasep®SF (DiaSys Ltd, Berkshire, England, DYS057-V14 04/2008) device is composed of a sedimentation cone, mixing chamber, Parasep lid and a filtration matrix. The device was assembled and sealed by fastening the filter thimble onto the sedimentation cone and mixing chamber. The tubes and sedimentation cones were labelled with the specimen identification codes. The Mini-Parasep® SF faecal concentrator was used per the manufacturer’s instructions as follows: the stool sample filled the spoon at the end of the filter. After adding 3.3 ml of 10% formalin and 0.1% Triton-X, the mixture was vortexed for 15 s. The Mini-Parasep® SF apparatus was then inverted and centrifuged at 1500 r/min for 2 min. The mixing chamber and filter were then unscrewed and discarded. After removing the supernatant fluid, the sediment was used for microscopic examination.
3. The Mini-FLOTAC apparatus comprises two main units, the base and the reading disc. There are two flotation chambers (1 ml each) which are designed for optimal examination of faecal suspensions. Fill-FLOTAC is a disposable device, which is part of the FLOTAC and mini-FLOTAC kits. It is used for the collection, homogenization and filtration of faecal samples (Cringoli 2006). Each faecal sample was processed using two floatation solutions of the Mini-FLOTAC concentrator: FS2 (saturated sodium chloride solution: specific gravity = 1.20) and FS7 (zinc sulphate solution: specific gravity = 1.35). The Mini-FLOTAC procedure was conducted according to Barda et al. (2013) protocol as follows: 2 g of fresh faeces was placed in the Fill-FLOTAC container. The faecal sample was then diluted in 2 ml of 5% formalin, thoroughly homogenized and filtered. Afterwards, 2 ml of the suspension (2 g of stool and 2 ml of formalin) was added to 36 ml of the FS2 and to 46 ml of FS7. The faecal suspension was then poured into the two floatation chambers of the Mini-FLOTAC. An average time of 10 min was required for the cysts and eggs to float before examining the reading disc (Barda et al. 2014).

Following the performance of different diagnostic techniques, slides were scanned under 10 × , 40 × and 100 × objectives and examined by two qualified medical parasitologists who were blind to the method used for sample preparation.

### Statistical analysis

Collected data were tabulated and analysed using IBM SPSS Statistics for Windows version 25. Descriptive statistics were used to report the prevalence that could be described as the proportion of positive samples over total samples analysed (Burning and Kintz 1997). The non-parametric chi-square (*χ*^2^) test was applied to check the statistical association between intestinal parasitic infection and the used variables (gender, residence, socioeconomic status and age groups). Significance was set at *P* values < 0.05, and 95% CI values were estimated. Logistic regression analyses were employed to determine independent risk factors for the infection (Bursac et al. 2008). According to Versi (1992), the term gold standard refers to the best available one that has a standard with known results. In the current study settings, this term best applied to the direct smear method.

*Ethical considerations.*

Before data or sample collection, all procedures were explained to the study participants and informed written or thumb-printed consents were obtained. Parents/guardians of the selected children also completed consent agreement forms. Patients were recognized by codes, as data were kept anonymous. The current study was also reviewed and approved by the Committee of Research, Publications and Ethics of the Faculty of Medicine, Zagazig University, Egypt. We certify that the study was performed in accordance with the ethical standards as laid down in the 1964 Declaration of Helsinki and its later amendments.

## Results

### Characteristics of the study population

In total, 500 patients participated in this study. Among them, 290 (58%) were males, and 210 (42%) were females. The age of the patients ranged from 11 to 60 years old (age mean ± SD = 31.478 ± 14.67). The distribution of patients by age groups was as follows: 11–20 years, 160 (32%); 21–40 years, 195 (39%); and 41–60 years, 145 (29%). More than half (57.6%) of the cases came from rural areas, and only 20% were of a high social status (Table 1). Out of the 500 patients, 105 (21%) were hospitalized (in-patients) and 395 (79%) were non-hospitalized (outpatients).

**Table 1:** Socio-demographic features of the study population (*n* = 500)

Characteristics	Category	Number	Percentage
Age groups	11–20 21–40 41–60	160 195 145	32% 39% 29%
Sex	Male Female	290 210	58% 42%
Residence	Rural Urban	288 212	57.6% 42.4%
Socioeconomic status (SES)	Low Medium High	205 195 100	41% 39% 20%
Crowding index	< 1 1–2 > 2	65 210 225	13% 42% 45%

### Prevalence of intestinal parasitic infections

**Fig. 1:**
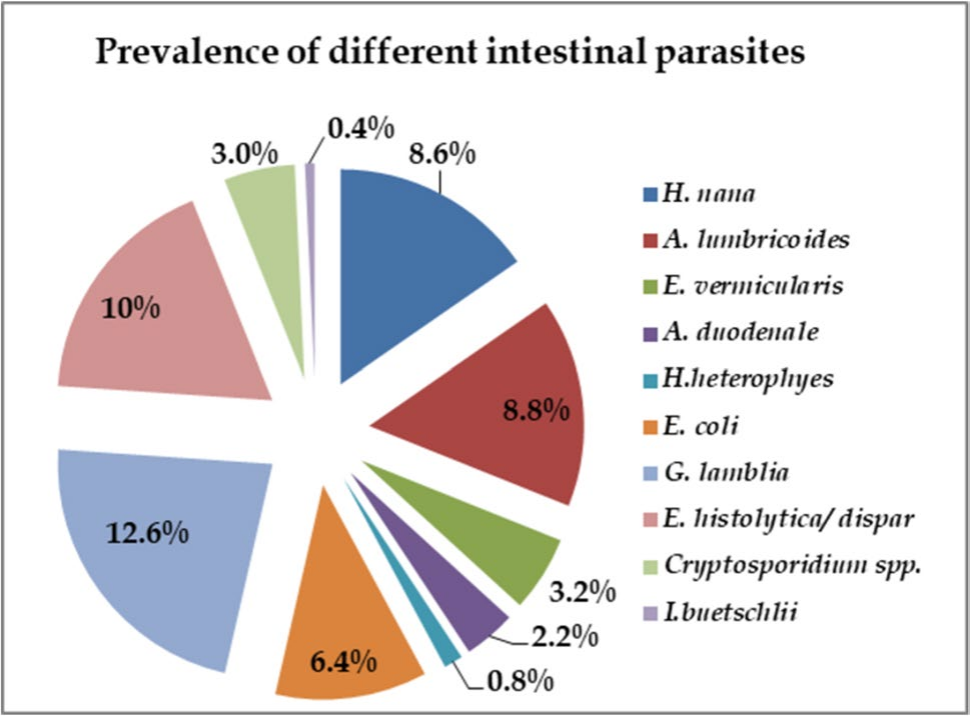
Prevalence of intestinal parasitic infections, by species, among 500 examined cases

Based on microscopic examination using the direct smear method, the overall prevalence of intestinal parasitic infections among the 500 examined cases was determined to be 56% (280/500). The prevalence of protozoan infections 32.4% (162/500) was higher than the helminth infections 23.6% (118/500). The most commonly detected protozoa were *Giardia lamblia* (*G. lamblia*) (12.6%), *Entamoeba histolytica/dispar* (*E. histolytica/dispar*) (10%) and *Entamoeba coli* (*E. coli*) (6.4%). While the most recovered helminths were *Ascaris lumbricoides* (*A. lumbricoides*) (8.8%), *Hymenolepis nana* (*H. nana*) (8.6%) and *Enterobius vermicularis* (*E. vermicularis)* (3.2%), the least recovered parasites during this study were *Iodamoeba buetschlii* (*I. buetschlii*) (0.4%), *Heterophyes heterophyes* (*H. heterophyes*) (0.8%) and *Ancylostoma duodenale* (*A. duodenale*) (2.2%) (Fig. 1).

According to Table 2, the prevalence of intestinal parasitic infections was significantly higher in the age group 11–20 years (65.6%, *P* < 0.05), while the other age groups 21–40 years and 41–60 years were less affected, with similar infection rates (51.3% and 51.7%, respectively). Males were found to have a higher percentage of infection (66.6%) compared with female subjects (41.4%). The prevalence of intestinal parasitism was significantly (*P* < 0.001) related to residence, socioeconomic status and the household crowding index (HCI). Rural residents had more frequent intestinal parasitic infections (64.9%) than urban residents (43.9%). Patients of low social classes and high crowding indices had the highest rates of infections (79.5% and 64.4%, respectively).

**Table 2:** The association between intestinal parasitic infections and potential risk factors among 500 examined cases

Risk factor	Category	Total (*n* = 500)	Positive cases (*n* = 280)		Negative cases (*n* = 220)		*χ*^2^	*P* value	OR (95% CI)
			No	%	No	%			
Age groups	11–20 21–40 41–60	160 195 145	105 100 75	65.6% 51.3% 51.7%	55 95 70	34.4% 48.7% 48.3%	8.85	0.01*	1.78 (1.12–2.83) 0.68 (0.64–1.51) Ref
Sex	Male Female	290 210	193 87	66.6% 41.4%	97 123	33.4% 58.6%	31.2	< 0.001**	2.81 (1.95–4.06)
Residence	Rural Urban	288 212	187 93	64.9% 43.9%	101 119	35.1% 56.1%	21.99	< 0.001**	2.37 (1.65–3.41)
Socioeconomic status (SES)	Low Medium High	205 195 100	163 84 33	79.5% 43.1% 33%	42 111 67	20.5% 56.9% 67%	80.68	< 0.001**	7.88 (4.6–13.49) 1.54 (0.92–2.54) ref
Crowding index	< 1 1–2 > 2	65 210 225	19 116 145	29.2% 55.2% 64.4%	46 94 80	70.8% 44.8% 35.6%	25.46	< 0.001**	Ref 2.99 (1.64–5.44) 4.39 (2.41–8)
Status of the patients	Hospitalized Non-hospitalized	105 395	54 226	51.4% 57.2%	51 169	48.6% 42.8%	1.13	0.29 NS	1.26 (0.82–1.94)

*χ*^2^ chi-square test, *OR* odds ratio, *CI* confidence interval. *Significant (*P* < 0.05), **highly significant (*P* < 0.001), *NS* non-significant (*P* > 0.05).

**Fig. 2:**
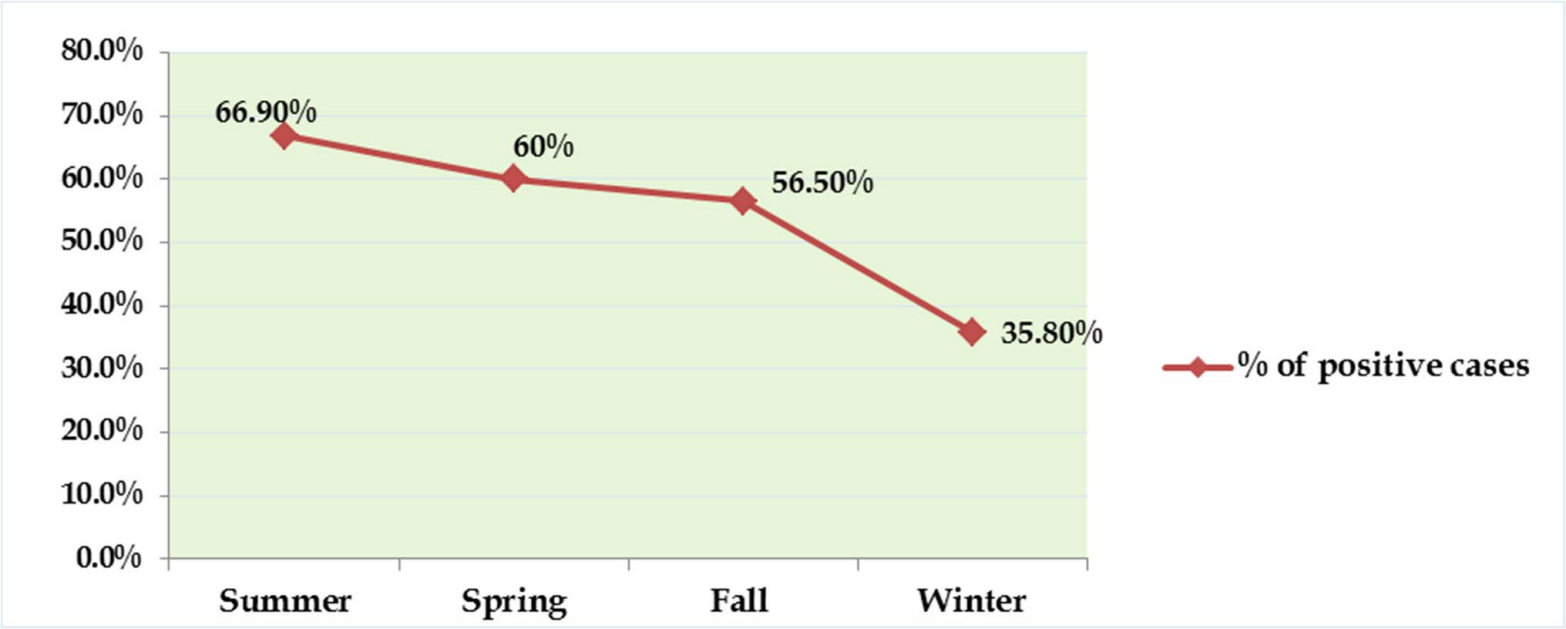
Distribution of intestinal parasitic infections during different seasons of study

Regarding the status of the patients, intestinal parasitic infections were more frequent in non-hospitalized cases with 57.2% than in hospitalized patients with 51.4%. However, the difference was statistically insignificant (*P* > 0.05) (Table 2). There was a significant variability (*P* < 0.001) in the distribution of parasitic infections in different seasons of study. The infection was highest in summer (66.9%) and spring (60%) and lowest in winter (35.8%) (Fig. 2).

### Factors associated with intestinal parasitic infections

Further analysis of data was performed to determine the possible risk factors that could be related to intestinal parasitic infections. In the logistic regression analyses, different socio-demographic factors like the age of the participants, 11–20 years (OR = 1.78; 95% CI, 1.12–2.83); male patients (OR = 2.81, 95% CI, 1.95–4.06); and rural residents (OR = 2.37; 95% CI, 1.65–3.41) were significantly associated with intestinal parasitic infections. Likewise, participants of low social class (OR = 7.88; 95% CI, 4.6–13.49) and high crowding index > 2 (OR = 4.39; 95% CI, 2.41–8) were identified as significant risk factors for intestinal parasitism. On the other hand, the prevalence of the infection was independent of the hospitalized/non-hospitalized status of the patients (OR = 1.26; 95% CI, 0.82–1.94) (Table 2).

Among the potential risk factors investigated during this study, seasonality was found to have a significant correlation with intestinal parasitism. There was a statistically significant increase in the frequency of the infection in summer (OR = 3.63; 95% CI, 2.16–6.09), spring (OR = 2.69; 95% CI, 1.58–4.58) and fall (OR = 2.33; 95% CI, 1.36–4) compared to winter (Table 3).

**Table 3:** Logistic regression analysis of intestinal parasitic infections during different seasons

Season	Total	Positive cases (*n* = 280)		Negative cases (*n* = 220)		χ^2^	*P* value	OR (95% CI)
		No	%	No	%			
Summer	151	101	66.9%	50	33.1%	26.18	< 0.001**	3.63 (2.16–6.09) 2.69 (1.58–4.58) 2.33 (1.36–4) Ref
Spring	125	75	60%	50	40%			
Fall	115	65	56.5%	50	43.5%			
Winter	109	39	35.8%	70	64.2%			

*χ*^*2*^ chi-square test, *OR* odds ratio, *CI* confidence interval. **Highly significant (*P* < 0.001).

### Comparison of commercial faecal concentrators in the recovery of different intestinal parasites

The diagnostic performance of the used commercial faecal concentrators in diagnosing different intestinal parasites, using direct smear as a gold standard test, is demonstrated in Table 4. Our results showed that the Mini-Parasep®(SF) procedure had the highest efficacy in the detection of different intestinal parasites. Its overall sensitivity, specificity and accuracy were 68.2%, 95.9% and 80.4%, respectively. The kit has also exhibited a higher sensitivity for helminths (72.9%) than for protozoan infections (64.8%). Regarding the two flotation solutions of the Mini-FLOTAC technique, FS2 showed a higher sensitivity than FS7 in the recovery of intestinal helminths (74.6% vs 53.4%), while FS7 was more sensitive in diagnosing intestinal protozoa (50.6% vs 43.8%) (Table 4).

**Table 4:** Diagnostic performance of commercial faecal parasite concentrators in the diagnosis of intestinal parasitic infections using direct faecal smear as a gold standard test

Parasitic infection	Calculated parameter	Mini-FLOTAC (FS2)	Mini-FLOTAC (FS7)	MP®SF
Overall	Sensitivity Specificity NPV PPV Accuracy	56.7% 91.8% 62.5% 89.8% 72.2%	51.7% 89.5% 59.3% 86.3% 68.4%	68.2% 95.9% 70.3% 95.5% 80.4%
Helminths	Sensitivity Specificity NPV PPV Accuracy	74.6% 93.8% 83.5% 89.8% 85.7%	53.4% 93.8% 73.4% 86.3% 76.8%	72.9% 97.5% 83.1% 95.6% 87.1%
Protozoa	Sensitivity Specificity NPV PPV Accuracy	43.8% 93.2% 54.7% 89.8% 64.6%	50.6% 88.9% 56.7% 86.3% 66.8%	64.8% 95.8% 66.5% 95.5% 77.9%

*MP® SF*, Mini-Parasep® solvent-free concentrator; *Mini-FLOTAC NaCl (FS2)*, Mini-FLOTAC ZnSO4 (FS7); *NPV*, negative predictive value; *PPV*, positive predictive value.

**Fig. 3:**
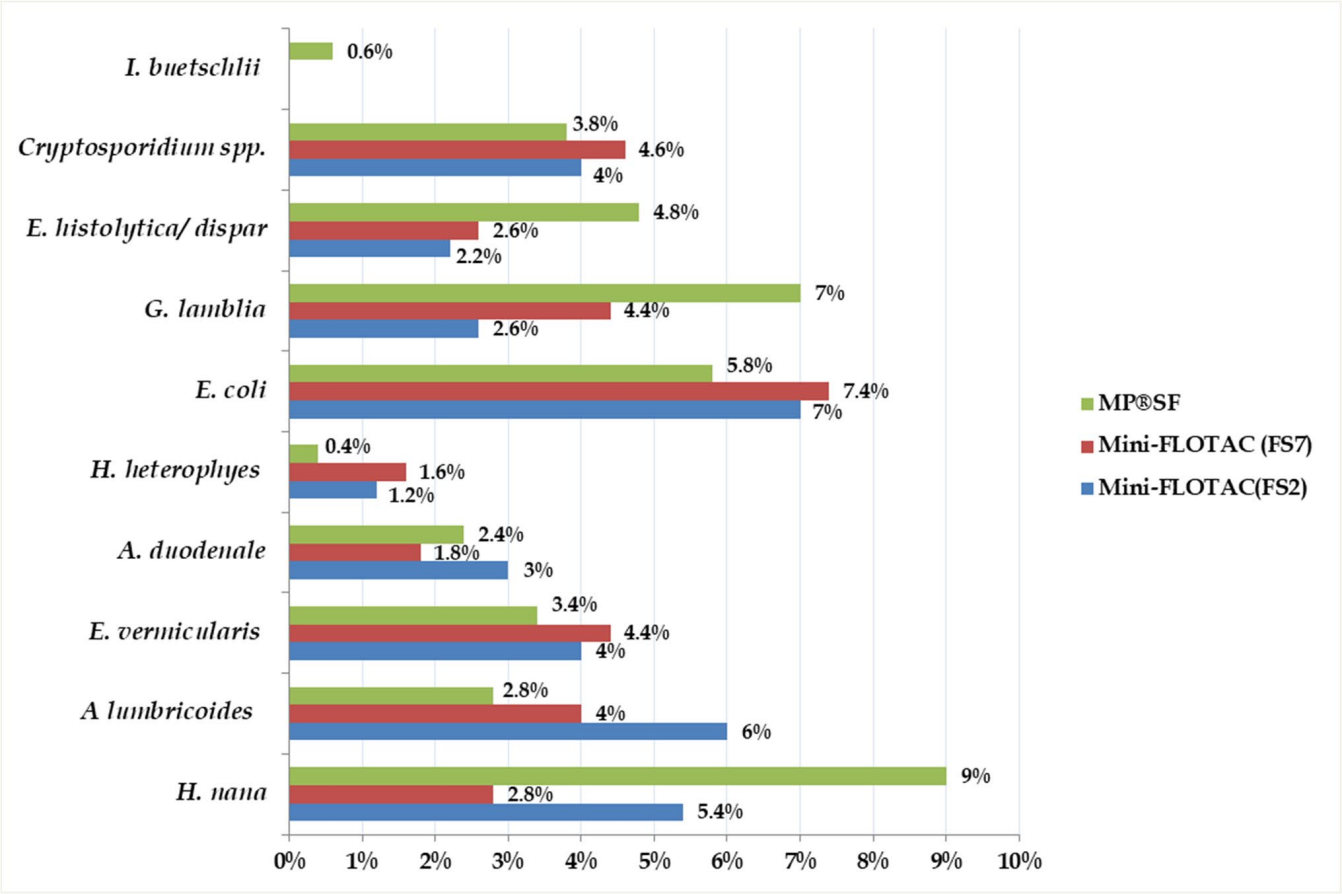
Positive detection rates of intestinal parasites determined by the commercial faecal parasite concentrators. Each bar of the chart represents the proportion of cases infected. MP®SF, Midi-Parasep®solvent-free concentrator; Mini-FLOTAC ZnSO4 (FS7); Mini-FLOTAC NaCl (FS2)

Positive detection rates of the recovered parasite species using faecal concentration kits are shown in Fig. 3. The Mini-Parasep® (SF) method showed higher infection rates for *H. nana* (9%), *G. lamblia* (7%) and *E. histolytica/dispar* (4.8%). Among the recovered parasite species, *I. buetschlii* cysts were found using only the Mini-Parasep®(SF) measure, which recorded a positivity of 0.6%, where the Mini-FLOTAC approach failed to detect any case (0%). As for geohelminths, more frequent infections were reported using the Mini-FLOTAC method. Infection rates for *A. lumbricoides* (6%) and *A. duodenale* (3%) were demonstrated using Mini-FLOTAC NaCl (FS2), while more *E. vermicularis* cases (4.4%) were diagnosed by Mini-FLOTAC ZnSO4 (FS7). *H. heterophyes-*infected patients were better diagnosed by the Mini-FLOTAC (FS7) measure, which detected 1.6% of the cases, while the Mini-Parasep®(SF) kit diagnosed only 0.4% of the cases (Fig. 3).

**Fig. 4:**
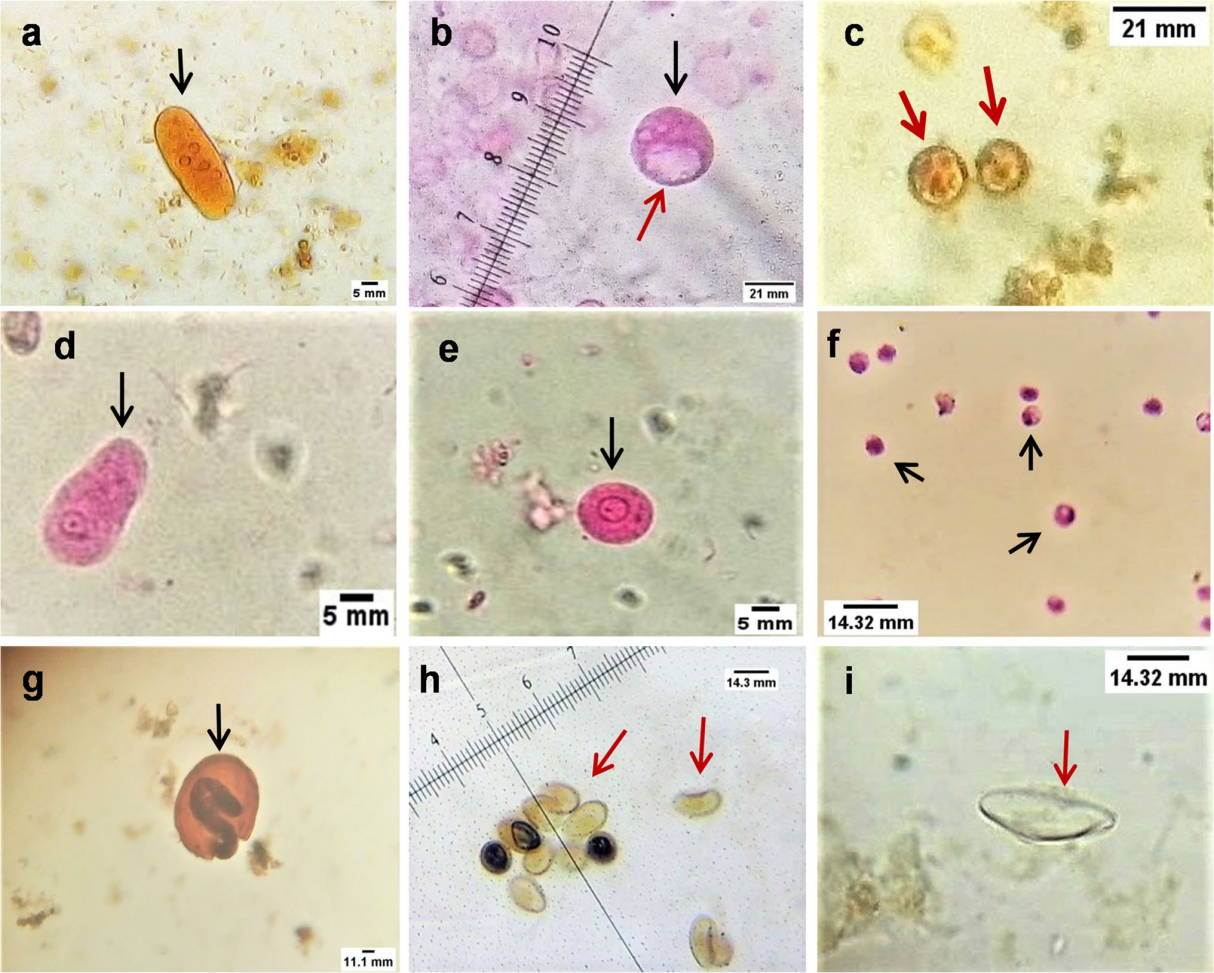
Photomicrographs of different intestinal parasites recovered during the study. **a** Elongated *E.coli* dividing trophozoite with 4 nuclei seen in the plane of focus (black arrow) (iodine stain, × 400). **b** *I. buetschlii* cyst (black arrow) with a large glycogen vacuole (red arrow) (eosin stain, × 1000). **c** *E. histolytica/dispar* cysts (red arrows) (iodine stain, × 400). **d** *E. coli* trophozoite (black arrow) (eosin stain, × 400). **e** *E. coli* pre-cystic stage with one nucleus seen in the plane of focus (black arrow) (eosin stain, × 400). **f** *Cryptosporidium* oocysts (black arrows) (MZN stain, × 1000). **g** *A. lumbricoides* embryonated egg containing second stage larva (black arrow) (iodine stain, × 400). **h** *H. heterophyes* eggs (red arrows) (unstained smear, × 400). **i** *E.vermicularis* egg (red arrow) (unstained smear, × 400). **a–c** Recovered parasite species after concentration by Mini-Parasep®(SF). **d–f** Recovered parasite species after concentration by Mini-FLOTAC ZnSO4 (FS7)*.* **g**, **h** Recovered parasite species after concentration by Mini-FLOTAC NaCl (FS2). **i** Recovered parasite in the unprocessed stool sample

Both protozoan and helminthic parasitic elements were recovered in stool samples. Photomicrographs of some intestinal parasites detected using different techniques are depicted in Fig. 4.

## Discussion

Parasitic infections constitute the greatest single worldwide cause of disease and illness. They cause critical health threats because of their morbid nature (Hailu and Ayele 2021). Intestinal parasitic infections affect millions of people around the world, particularly in developing countries (PAHO 2019). Reliable and practical diagnostic measures are essential for the proper evaluation of the prevalence of intestinal parasitism. Understanding the distribution pattern of intestinal parasitic infections is a crucial step for planning efficient intervention programmes to improve the health status (McCarthy et al. 2012). The present study assessed the current situation of human intestinal parasitism among patients attending two major teaching hospitals in Zagazig city, Northeastern Egypt.

In the present study, 56% of the examined cases were positive for different intestinal parasitic infections. This finding was consistent with Yones et al. (2019), who reported a 56.3% prevalence rate of gastrointestinal parasites among Egyptian school children in rural Assiut Governorate. Yet, a considerable variation in the overall prevalence of intestinal parasitism was observed by comparing our results to those already recorded in different regions of Egypt. This variation could be explained by the group of patients examined. In the group of food handlers, Badawey et al. (2015) reported an overall prevalence of 32.4% for enteric parasitic infections among food handlers in Sharqia Governorate. Also, Yimam et al. (2020) recorded prevalence estimates of 33.6% for intestinal parasites among food handlers in Ethiopia. In the group of school children, Abdel Fatah and Nofal (2012), as well as Mahmoud et al. (2017), recorded prevalence rates of 33.6% and 37% among school children in Alexandria and Dakhalia Governorates, respectively.

In terms of the detected intestinal parasites, the most commonly identified species were *G. lamblia* (12.6%), *E. histolytica/dispar* (10%), *A. lumbricoides* (8.8%) and *H. nana* (8.6%) (Fig. 1). These results come in accordance with previous studies conducted inside Egypt. Farghly et al. (2016) reported *G. lamblia* (26%) and *E. histolytica* (13.4%) as the most prevalent parasitic infections among school children in Zagazig district, Northeastern Egypt. Likewise, Hussein et al. (2017) described *G. lamblia* as the most common parasitic infection (26%) among patients with gastrointestinal troubles in the Greater Cairo region, Northern Egypt. The similarity between the parasitic species recovered during the present research and other comparable studies conducted in Egypt could be attributed to the fact that the Egyptians might be subjected to the same risk factors, which include consumption of unwashed vegetables, ineffective water treatment systems and poor hygienic habits.

Our results were also endorsed by different studies outside Egypt. Baral et al. (2017) recorded *G. lamblia* (3.34%) followed by *E. histolytica/dispar* (1.96%) as the most prevalent parasitic infections in a tertiary care hospital in Nepal. Similarly, *E. histolytica* (5.5%) followed by *A. lumbricoides* (4%) and *G. lamblia* (3%) were reported as the dominant parasites detected among asymptomatic food handlers in Ethiopia (Kebede et al. 2019).

Among different helminth species detected during this study, *A. lumbricoides* was the most frequently recovered parasite (8.8%), followed by *H. nana* (8.6%) (Fig. 1). The obtained results were supported by a recently conducted study in Kafr El-Sheikh Governorate, Northwestern Egypt. In their research, Allam et al. (2021) found that *Schistosoma mansoni*, *H. nana* and *A. lumbricoides* were the most prevalent helminthic infections among the primary school children of the Governorate. In the current work, *A. duodenale* was one of the least recovered parasites (2.2%) (Fig. 1). Yet, this finding contradicts Yones et al. (2019), who showed that *A. duodenale* (8.7%) was one of the dominant parasitic infections among rural school children in Assiut Governorate, Southern Egypt. Recent studies have demonstrated a rise in the prevalence rates of hookworm infections. Eyayu et al. (2021) identified hookworm species (13.3%) as the second leading cause of intestinal parasitosis among patients attending a primary hospital, Northwestern Ethiopia. Also, Taghipour et al. (2021) recorded high levels of hookworm infections (80.7%) among pregnant females.

The results of the current study revealed a high prevalence of intestinal parasitic infections among the 11–20 years age group (65.6%) (Table 2). This finding was endorsed by Amer et al. (2018) who attributed the high rate of infection in this age category to their close contact with the contaminated environment. Regarding the gender, this study reported significantly (*P* < 0.001) higher infection rates in males (66.6%) compared with female cases (41.4%) (Table 2). Similar observations were demonstrated by other studies inside and outside Egypt. El-Sherbini and Abosdera (2013) recorded higher infection rates of intestinal parasites among male children in Giza Governorate, Egypt. Also, Hailu and Ayele (2021) have demonstrated more positive parasitic infections among male school children in Debre Berhan town, Northeast Ethiopia. However, Gelaw et al. (2013) and Diongue et al. (2017) reported a higher frequency of intestinal parasitic infections among female subjects.

The relationship between the prevalence of parasitic infections and either age or gender of the study population remains controversial. Our study demonstrated a strong association of intestinal parasitic infections with both the age and gender of the study participants (Table 2). Similarly, Akinbo et al. (2011), as well as Hailu and Ayele (2021), reported age as a potential risk factor for intestinal parasitism. Moreover, Hassen Amer et al. (2016) have reported a significant relationship between gender and intestinal parasitic infections. On the other hand, Hussein et al. (2017) and Abbaszadeh Afshar et al. (2020) denied any correlation between either age or gender and intestinal parasitism. We have also shown that intestinal parasitosis was dependent on the type of residence, socioeconomic status and the household crowding index (Table 2). This finding was compatible with Geneidy (2019), who reported that low social classes were significant risk factors for intestinal parasitic infections. Likewise, Ngui et al. (2011) confirmed the influence of low household purchasing power on intestinal parasitosis in Malaysia. Conversely, Almeida et al. (2017) recorded that low family income was not significantly associated with intestinal parasitic infections.

Regarding the hospitalized/non-hospitalized status of the patients, we have denoted that, despite the statistically insignificant difference (*P* = 0.29), intestinal parasitic infections were more frequent among non-hospitalized cases (57.2%) (Table 2). This same observation was supported by Diongue et al. (2017). Our study has reported the highest prevalence rates of the infection during the summer months (66.9%) and the lowest infection rates in winter (35.8%) (Fig. 2). Additionally, Amer et al. (2018) have denoted the relationship between seasonality and intestinal parasitic infections. They recorded a high level of infection in fall (0.79%) and summer (0.63%) seasons and a lower prevalence during winter (0.25%). The current study reported a significant (*P* < 0.001) seasonal variation in the prevalence of intestinal parasitic infections (Table 3). However, this finding disagrees with Akinbo et al. (2011), who demonstrated that intestinal parasitism was not significantly related to different seasons.

Accurate diagnosis of parasitic infections is critically important for both population-based studies and individual patient management. The choice of a single (gold standard) test for diagnosing intestinal parasitism is hard to achieve. According to Momčilović et al. (2019), direct microscopic stool examination remains the cornerstone of parasitological diagnosis. Furthermore, Mbong Ngwese et al. (2020) regarded the direct smear measure as an essential technique for detecting different parasitic elements in a faecal sample. Therein, our study tried to assess the intestinal parasitic situation in the city of Zagazig using two innovative faecal parasite concentrators (Mini-Parasep®SF and Mini-FLOTAC), with the direct smear measure as a gold standard. In the present study, the calculated analytical sensitivity, specificity and accuracy (68.2%, 95.9% and 80.4%, respectively) indicated that the MP®SF kit was the most effective tool in diagnosing different intestinal parasites. The faecal concentrator has also exhibited the highest diagnostic accuracy (87.1%) in detecting intestinal helminths (Table 4). This finding was supported by Ng’etich et al. (2016), who approved the diagnostic potential of Mini-Parasep as a promising alternative tool in diagnosing and monitoring schistosomiasis transmission. They denoted that the Mini-Parasep technique displayed a higher sensitivity (77.5%) than the standard Kato-Katz measure (33.8%) for detecting *Schistosoma mansoni* infections.

In comparison with the Mini-FLOTAC procedure, Mini-Parasep®SF had a higher sensitivity (64.8%) in diagnosing intestinal protozoa (Table 4). The highest positive detection rates of *G. lamblia* (7%), *E. histolytica/dispar* (4.8%) and *I. buetschlii* (0.6%) were obtained using the Mini-Parasep®SF technique (Fig. 3). Similar observations were achieved by Abdel Aziz et al. (2020), who also recorded a high sensitivity (83.3%) for the Midi-Parasep® kit in diagnosing intestinal protozoa, particularly *E. histolytica/dispar* and *G. lamblia.* Also, Mohram et al. (2020) have described Mini-Parasep® SF as the most effective method for detecting *Giardia* cysts. In their study, more than 80% (82.7%) of *G. lamblia-*infected cases were diagnosed using the Mini-Parasep procedure.

In the current study, two floatation solutions were used for each Mini-FLOTAC procedure. The first was NaCl (FS2) solution, which has shown the highest sensitivity in the recovery of helminth infections (74.6%). The other was ZnSO4 (FS7) solution, which was more sensitive than FS2 in diagnosing intestinal protozoa (50.6% vs 43.8%) (Table 4). Compared with the Mini Parasep®SF technique, the study has also shown a superior efficacy of the Mini-FLOTAC kit in detecting geohelminths. The highest positive detection rates of *A. lumbricoides* (6%), *A. duodenale* (3%) and *E. vermicularis* (4.4%) were obtained using the Mini-FLOTAC approach (Fig. 3). The efficacy of the Mini-FLOTAC technique in detecting helminths infections has been confirmed in several studies. Levecke et al. (2009) recorded that the FLOTAC measure turned out to be the most sensitive approach (95%) for helminth diagnosis. Barda et al. (2013) have also reported that the Mini-FLOTAC technique was the most sensitive measure (90%) for diagnosing intestinal helminths, compared to only 30% sensitivity for the direct smear method. Likewise, El-Nadi et al. (2019) reported a 100% sensitivity of the Mini-FLOTAC approach in diagnosing helminth infections. Our study has also reported a relatively low diagnostic accuracy of the Mini-FLOTAC ZnSO4 (FS7) in diagnosing intestinal protozoa (66.8%) (Table 4). However, this finding opposes Allam et al. (2021), who recorded a marked diagnostic accuracy of the FLOTAC procedure (99%) in diagnosing intestinal protozoan infections among rural Egyptian school children.

## Conclusions

In conclusion, the present study revealed a high prevalence (56%) of intestinal parasitic infections among patients attending two major hospitals in Zagazig district, which indicates the public health importance of these infections. The study has identified low ages, male patients, rural residents, low social classes and high crowding indices as potential risk factors for intestinal parasitosis. The study has also shown the diagnostic accuracy of the Mini-Parasep®SF concentrator in diagnosing both helminth and protozoan infections, whereas Mini-FLOTAC (FS2) has exhibited the highest sensitivity for helminths recovery. Till now, little attention has been paid towards using different approaches for mass diagnosis under field conditions. Hence, the authors recommend combining the two innovative faecal parasite concentrators (Mini Parasep®SF and Mini-FLOTAC) as promising alternative tools in routine laboratory and community-based diagnosis of intestinal parasitic infections.

## Data Availability

All data and materials used to support the findings of this study are included within this article.
